# Niche Suitability Evaluation and Path Selection for the High-Quality Development of Cities in the Yellow River Basin

**DOI:** 10.3390/ijerph20043727

**Published:** 2023-02-20

**Authors:** Peizhe Shi, Zhaohan Lu, Mengqing Zhou, Ning Wang, Yuping Wu

**Affiliations:** Research Center of Energy Economics, School of Business Administration, Henan Polytechnic University, Jiaozuo 454003, China

**Keywords:** niche theory, watershed urban development, suitability path, Yellow River basin

## Abstract

The urban development in the Yellow River basin (YRB) varies widely. Therefore, it is necessary to choose a development path that fits the characteristics of each city to achieve high-quality development. The purpose of this paper is to address the problem of how to choose a characteristic path for high-quality development and clarify its suitability for YRB cities. Firstly, based on data from 50 YRB cities from 2011 to 2020, the suitability evaluation was carried out from the perspective of an ecological niche, followed by the measurement of sub-dimensional niche breadth and overlap. The results confirmed the great diversity of development between cities and the intense competition for resources. Then, based on the classification approach using the k-means method, this study proposes a method for selecting a suitable path for high-quality development. It classifies the suitable paths into 3 major types with 7 minor types and recommends policies for the suitable paths for YRB cities. The systematic thinking and specific path selection method for the high-quality development of YRB cities is not only of practical significance for implementing city classification strategies but also provides a reference for the sustainable development of basin cities in other countries.

## 1. Introduction

The Yellow River basin (YRB) covers an area of 795,000 square kilometers, spanning 9 Chinese provinces within 60 cities, accounting for 8.3% of China’s land area [1,2]. As the mother river of the Chinese people, the populations living in the cities through which the watershed flows account for 30% [3], while the regional GDP accounts for a quarter of China’s total. In 2017, the Chinese government proposed the ecological protection and high-quality development of the YRB as major strategic goals for China’s national development [4,5]. However, the YRB covers many cities with different development bases and resource endowments. Therefore, it is necessary to investigate high-quality development paths for cities in the YRB. To promote high-quality development in the YRB, the Chinese government expects each locality to actively investigate a unique path for high-quality development enriched with regional characteristics. Applying appropriate strategies that are suitable for the local reality and timely conditions in accordance with the principle of adapting to local conditions is the key to achieving high-quality development in the YRB [6].

Previous scholars have conducted fruitful research studies on the high-quality development of the YRB, focusing on two aspects, for example, the level of high-quality development. As research progressed, scholars began to explore quantitative methods to analyze the weaknesses of high-quality development in the basin, among which the measurement of the high-quality development level was the earliest area of concern. Some scholars have constructed a measurement system of high-quality development in the YRB based on the new development concept [7,8,9]. For example, Ren et al. evaluated the status of high-quality economic development in 9 provinces and regions of the YRB and each main functional area based on innovation, coordination, green, openness, and sharing dimensions [10]. Other scholars have measured the level of high-quality development in the YRB based on the economic-social-ecological dimensions [11]. For example, Shi et al. measured the level of high-quality economic development of 77 cities above the prefecture level in the YRB from 3 dimensions, i.e., fundamentals of development, social outcomes, and ecological outcomes [12]. On the other hand, there are studies on high-quality development paths and implementation strategies. Some scholars studied the high-quality development of the YRB from the perspective of the basin as a whole [13], and argued that the high-quality development of the YRB should be based on ecological protection [14], focusing on the development of scientific and technological innovation [15,16], legislation [17], and collaborative governance [18]. Some other scholars believe that the high-quality development of the YRB should be studied by zoning and classification [19], and the studies focus on high-quality development in industries [20], cities [21], and specific regions [22]. Existing studies have been conducted from different perspectives and dimensions to provide theoretical support and policy recommendations for promoting high-quality development in the YRB. However, most of the current studies on the path of high-quality development are qualitative studies, and there is no consensus and scientific method for classifying cities in the basin, and, thus, they lack academic and rational classification analyses and the basis of policy application.

From the perspective of ecology, the region through which the Yellow River flows is a special natural–economic–social composite ecosystem, and its high-quality development is constrained and limited by several factors, such as natural resource endowment and infrastructure. Each city in the basin occupies different resources and is in a different niche in this ecosystem, and has different paths and strategies for its development. The ecological niche concept in ecology was first defined by Grinnell, who considered ecological niche as “the ecological space occupied by an individual or a population” [23]. Whittaker further extended the concept of ecological niche, defining it as the position of a species in its environment and its interrelationship with other species [24]. With the depth of research, the ecological niche theory has been widely used in the study of inter-species relationships, population evolution, biodiversity, and stability, and applied in the fields of regional development and industrial development [25,26,27]. For example, Ji used the ecological niche theory to study the evolution of supply chain clusters [28], while Huang evaluated the socioeconomic–ecological complex ecosystem of Chengdu–Chongqing urban agglomeration by constructing an index system containing the resource niche, the environmental niche, the economic niche, and the social–ecological niche [29]. Therefore, the ecological niche theory helps one to analyze more intuitively the positioning of the high-quality development of YRB cities and clarify the suitable development paths for cities. However, the application of ecological niche theory in the field of high-quality development, especially at the city level, is relatively rare, and the relevant laws are still unclear and need to be further explored.

Based on the research background analysis and literature review, this study argues that the high-quality development of cities in the YRB starts from the actual current situation of the overall basin, aims to solve the current main contradictions of cities in the YRB, and determines the all-around development of suitable development paths by combining the regional resource endowments. Based on factor endowment and ecological niche theory, this paper explores a suitability evaluation system that can comprehensively and accurately assess the environment and conditions for high-quality development in YRB cities. The suitability development of YRB cities is evaluated and measured using ecological niche-related theories and models, and then the K-means classification method is applied to classify the evaluation and measurement results; the selection methods of suitability paths for high-quality development are proposed on the basis of the classification results. In this way, this study attempts to address the problems of “how to choose”, “what to choose”, and “how to do” the paths of high-quality development of YRB cities. This will provide a theoretical basis and policy recommendations for the classification of the YRB to achieve high-quality development.

The main innovations of this study are as follows: (1) An index system for evaluating the niche suitability of the high-quality development of cities in the YRB is created based on the ecological niche theory and the factor endowments of the cities in the YRB, in which the 7 sub-dimensional innovatively reflect the reality of the resource elements on which the cities’ development depends. This makes the process of evaluating the high-quality development more refined and the results more intuitive. Thus, it clarifies the resources and competitive advantages of each city in the basin from the perspective of sub-dimensional ecological niche, and provides a basis for finding the path that fits their own development characteristics. (2) In the method of selecting suitable paths, we propose a suitable path classification method based on the niche suitability evaluation results, which classify the high-quality development paths of cities in the YRB into 3 major types (including 7 minor types): (1) the leading development path (including the central-leading path and regional-leading path); (2) the characteristic development path (including the agriculturally featured development path, industrially featured development path, service-featured development path, and ecological functional path); (3) the livelihood development path. The suitability path selection method allows this study to scientifically and feasibly identify the suitable paths for the high-quality development of cities in the YRB, referencing other basin cities domestically and internationally.

## 2. Methods and Materials

### 2.1. Study Area and Data Source

The current research focuses on the prefecture-level cities located in the YRB watershed. To facilitate the study, the selection of the research objects was based on the criteria of the classification of cities in the basin by Yang et al. A city is considered a YRB city if one of the following 3 criteria is fulfilled [30]: (1) More than half of a city’s area is in the YRB. (2) The main stream of the Yellow River or its first-class tributaries flow through this prefecture-level city. (3) The location of the government of a prefecture-level city is in the YRB.

**Figure 1:**
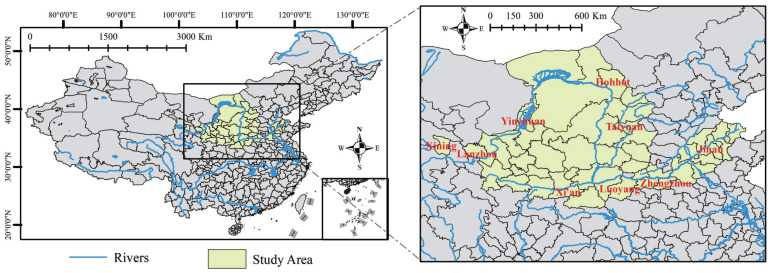
Map of of cities in the Yellow River Basin (YRB) in the current research.

Based on the above criteria, a total of 60 prefecture-level cities (states/alliances) distributed in 9 provinces were selected. Due to missing data on individual cities, 50 cities in the basin were finally studied in this study. The map of YRB cities is shown in Figure 1.

As shown in Figure 1, from the geographical point of view, the 50 cities in the YRB selected for the current research cover the upper, middle, and lower reaches of the YRB in terms of basin distribution, covering all major cities in the basin as shown in the above figure on the right. From the geographical distribution characteristics, it can be seen that the economic and social development levels of the 50 cities studied in the current research are representative of the current development levels of the YRB as a whole. These YRB cities have different development levels due to their different development bases. Therefore, it is meaningful to explore the suitable development path selection for YRB cities according to the development variations.

After determining the study area, according to the evaluation index system established in Section 2.2.1, this study collected relevant data for the above 50 cities during 2011–2020. The data of this study were obtained from official data, such as the China City Statistical Yearbook [31], the China Statistical Yearbook and the statistical bulletin of national economic and social development of each city [32]. To ensure the availability and reliability of the data, the missing value estimation method was used to supplement the missing data on individual cities in individual years.

### 2.2. Methods

**Figure 2:**
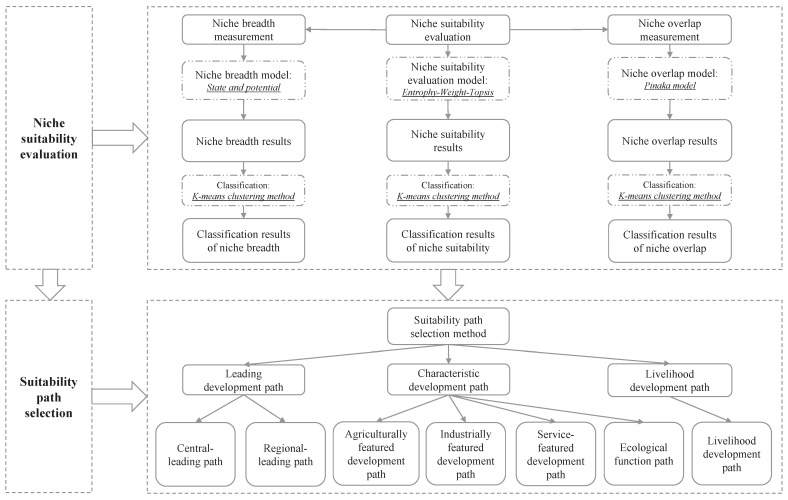
Flowchart of the study structure.

The study on the niche suitability evaluation and path selection for the high-quality development of cities in the YRB involves a combination of theoretical foundations and methodological innovations that are generalizable and replicable. Figure 2 shows the flow chart of the study structure.

Figure 2 shows the niche suitability evaluation of the high-quality development of basin cities from 2011 to 2020. Based on the evaluation results, the niche breadth and the niche overlap in the watershed cities are measured. Finally, the current research proposes a selection approach of suitable paths that integrates the above niche evaluation results.

#### 2.2.1. Niche Suitability Evaluation Model

In this study, the niche suitability of high-quality development refers to the suitability level of each ecological factor for the high-quality development of cities in the YRB, reflecting the resources of each sub-dimension and the relative position of a city in its environment for high-quality development. When evaluating the niche suitability of the high-quality development of cities in the YRB, this study first constructs a niche suitability index system for high-quality development. Then, the suitability values of sub-dimensional niche of each city is measured by using the niche suitability model. Finally, the results are rationally classified according to the measurement results and classification methods.

First, this paper constructs a niche suitability index system to evaluate the high-quality development of cities in the YRB. Combined with related research studies, this paper defines the niche of YRB cities as the sum of various resources, their economic and social functions, and relationships required for the high-quality development of YRB cities at certain times and spaces.

Based on the above definition and combined with the niche classification in related research [33,34], combined with the instructions of the Outline of the Plan for Ecological Protection and High-Quality Development of the YRB issued by China in 2021 [35], it is necessary to begin with the actual situation of each place; to cultivate economic growth poles, create open channel hubs, and drive high-quality development of the whole basin, this paper divides the high-quality development of the YRB into 7 sub-dimensional resources, i.e., infrastructure resources, service resources, openness resources, innovative resources, industrial resources, agricultural resources and ecological environment resources. Infrastructure resources refer to the system of public services used to ensure the socioeconomic activities required by the basin cities. Service industry resources consist of the sum of operations that use equipment, information, skills, etc., to provide labor and services to the basin city. Openness resources refer to the conditions and material bases required for the opening of the basin city to the outside world. Innovative resources refer to the various inputs needed for technological innovation in the basin city. Industrial resources refer to the sum of resources that directly or indirectly provide raw materials or power for industrial production. Agricultural resources refer to the sum of agricultural natural resources and socioeconomic factors that directly or indirectly play a role in agricultural production. Ecological environment resources refer to the series of material bases and real conditions that can be used in the process of development and utilization of ecological and environmental resources and their own cycles. These 7 resource dimensions interact with each other and are indispensable components for the high-quality development of watershed cities. Following the principles of systematicity and scientific data availability, this paper selects 25 evaluation indices that can reflect the high-quality development statuses of cities in the basin.

Table 1 shows the evaluation index system of the niche suitability for high-quality development in YRB cities.

After constructing the index system for evaluating the niche suitability of the high-quality development in the YRB, the entropy-weighted TOPSIS-integrated evaluation method was adopted to calculate the niche suitability; the specific steps are as follows [36,37,38]:

First, the raw data are standardized to obtain the normalization matrix $Z_{ij}$.

Let $X_{ij}\left(i=1,2,3,4,\ldots ,n,j=1,2,3,4,\ldots ,m;n=50,m=25\right)$, the observation of the *j*th index of the *i*th city value, create matrix $X=\left[\begin{array}{ccc} x_{11} & \ldots & x_{1m}\\ \ldots & \ldots & \ldots \\ x_{n1} & \ldots & x_{nm} \end{array}\right]$. Standardize $X_{ij}$ to obtain $Z_{ij}$:

(1) $$\begin{array}{c} Z_{ij}=\frac{x_{ij}-\min\left(x_{ij}\right)}{\max\left(x_{ij}\right)-\min\left(x_{ij}\right)}, \end{array}$$

$Z_{ij}$ is the positive index.

(2) $$\begin{array}{c} Z_{ij}=\frac{\max\left(x_{ij}\right)-x_{ij}}{\max\left(x_{ij}\right)-\min\left(x_{ij}\right)}, \end{array}$$

$Z_{ij}$ is the negative index.

Second, calculate the information entropy of each index.

(3) $$\begin{array}{c} P_{ij}=\frac{Z_{ij}}{\sum_{i=1}^{n}Z_{ij}} \end{array}$$

(4) $$\begin{array}{c} e_{j}=\frac{1}{\ln m}\sum_{i=1}^{m}p_{ij}\ln\left(p_{ij}\right) \end{array}$$

Third, calculate the weight coefficient $W_{j}$ of the *j*th index.

(5) $$\begin{array}{c} W_{j}=\frac{\left(1-e_{j}\right)}{\sum_{j=1}^{m}\left(1-e_{j}\right)} \end{array}$$

Fourth, calculate the Euclidean distance $D_{i}^{+}$ and $D_{i}^{-}$. Determine the positive and negative ideal solutions $Q_{i}^{+}$ and $Q_{i}^{-}$ of the city, set $Q_{i}^{+}$ as the maximum value of the *i*th city in the *j*th indicator among the objects, i.e., the positive ideal solution. Set $Q_{i}^{-}$ as the minimum value of the *i*th city in the *j*th indicator among the objects, i.e., the negative ideal solution. After determining the weight coefficient, use the TOPSIS method to select the decision-making plan. Establish the weighted matrix $r_{ij}$ of the evaluation indices for the high-quality development of YRB cities.

(6) $$\begin{array}{c} r_{ij}=W_{j}*Z_{ij} \end{array}$$

(7) $$\begin{array}{c} D_{i}^{+}=\sqrt{\sum_{j=1}^{m}{\left(Q_{j}^{+}-r_{ij}\right)}^{2}} \end{array}$$

(8) $$\begin{array}{c} D_{i}^{-}=\sqrt{\sum_{j=1}^{m}{\left(Q_{j}^{-}-r_{ij}\right)}^{2}} \end{array}$$

Finally, calculate the relative proximity $C_{i}$ of each *i* city to the ideal solution by using Formula (9). According to the score of $C_{i}$, the pros and cons of the schemes can be evaluated.

(9) $$\begin{array}{c} C_{i}=D_{i}^{-}/\left(D_{i}^{+}+D_{i}^{-}\right) \end{array}$$

#### 2.2.2. Niche Breadth Model

The niche breadth in the current research refers to the collection of all ecological factors possessed or available for high-quality development in YRB cities. The wider the niche breadth, the greater the diversity of resources utilized by the city, and the greater the demand for that dimension of resources for high-quality development. Similarly, the narrower the niche breadth, the more constrained the city’s development is.

Niche breadth is generally measured using methods such as Levins, the Shannon–Wiener niche breadth index, and the niche theory of state and potential. In this study, the niche breadth is measured using a combination of state and potential. The “state” describes the reality for high-quality development in YRB cities. Here, the niche suitability evaluation values of 50 YRB cities in 2015 and 2020 are measured. The “potential” describes the future development trend for high-quality development in YRB cities. In this paper, the increases of the niche suitability evaluation values from 2011 to 2015 and from 2016 to 2020 are used as the “potential”. The “potential” is measured on a time scale of n years and the dimension conversion coefficient is 1/n. Therefore, the dimension conversion coefficient for this study is 0.25. Thus, the niche breadth is calculated as follows [39].

(10) $$\begin{array}{c} N_{i}=\frac{S_{i}+A_{i}P_{i}}{\sum_{j=1}^{n}\left(S_{j}+A_{j}P_{j}\right)} \end{array}$$

In the formula: $S_{i}$, $P_{i}$ are the “state” and “potential” of city *i* and $S_{j}$, $P_{j}$, are the “state” and “potential” of city *j*. $A_{i}$, $A_{j}$ are dimension conversion coefficients; *n* is the number of cities. $\left(S_{i}+A_{i}P_{i}\right)$ is the absolute ecotone of city *i*, and $N_{i}$ is the niche breadth of city *i*.

#### 2.2.3. Niche Overlap Model

Niche overlap refers to the similarity of the high-quality development niche of any two cities in the YRB, which includes a measure of the similarity in the ability to utilize the same ecological factor. Using the niche overlap model, we can observe the similarities and differences in resource utilization in the YRB cities. In this study, the Pianka model was used to calculate the degree of niche overlap [40,41], and the calculation formula is as follows:

(11) $$\begin{array}{c} D_{ikj}=\frac{\sum_{j=1}^{n}F_{ij}F_{kj}}{\sqrt{\left(\sum_{j=1}^{n}F_{ij}^{2}\right)\left(\sum_{j=1}^{n}F_{kj}^{2}\right)}} \end{array}$$

In the formula, $D_{ikj}$ is the niche overlap value of cities *i* and *k* on resource *j*; the domain value is [0, 1]. $F_{ij}$ is the proportion of the resource utilization status of city *i* to the *j*th indicator to its utilization of the total resource status. $F_{kj}$ is the proportion of the resource utilization status of city *k* to the *j*th indicator to its utilization of the total resource status. The larger the value of $D_{ik}$, the greater the development mode between city *i* and city *k*. The larger the value of $D_{ik}$, the closer the development mode between city *i* and city *k*, and the higher the potential competition degree between them.

#### 2.2.4. Suitability Path Selection Method

The K-means clustering method is applied to classify the results of niche suitability, niche breadth, and niche overlap measures for high-quality development in YRB cities in a reasonable way. The K-means clustering method is an objective dynamic clustering method [42]. The method divides the results into K classes by cohesive points by viewing each sample equally and iterates continuously on the basis of the initial classification to make the final classification results more scientific [43]. In this paper, the results of niche suitability, niche breadth, and niche overlap for high-quality development in YRB cities are divided into 4 levels, which are assigned the values of 4, 3, 2, and 1 in the path selection method. The 4 levels of niche suitability are very suitable, relatively suitable, generally suitable, and unsuitable, which are assigned 4, 3, 2, and 1, respectively in the suitability path selection method; the 4 levels of niche breadth are wide, relatively wide, relatively narrow, and narrow, which are assigned 4, 3, 2, and 1, respectively, in the suitability path selection method; the 4 levels of niche overlap are high, relatively high, relatively low, and low, which are assigned 4, 3, 2, and 1, respectively, in the suitability path selection method. In order to make the results more intuitive, this paper uses the ArcMap application in ArcGIS software to visualize the results [44].

Based on the K-means clustering method, this paper proposes a path selection method for the suitability of high-quality development. The niche suitability, the niche breadth, and the niche overlap in the YRB cities are classified into four levels, and the four levels are assigned values according to the criteria stated above; the larger the value, the stronger the degree. Thus, the suitability path selection criteria for the high-quality development of 50 YRB cities were obtained.

The suitability paths for high-quality development in YRB cities are divided into 3 categories (including 7 subcategories). The selection criteria are as follows:

The first major path is the leading development path.

- Central-leading path:
  The number of niche sub-dimensions with niche suitability levels of 4 are greater than or equal to 1, the number of niche sub-dimensions with niche overlap levels of 4 are less than or equal to 4, and the number of niche sub-dimensions with niche breadth levels of 4 are greater than or equal to 3.
- Regional-leading path:
  Under the premise of considering location conditions, the number of niche sub-dimensions with niche suitability level greater than or equal to 2 are greater than or equal to 1, the number of niche sub-dimensions with niche overlap levels of 3 are less than or equal to 2, and the number of niche sub-dimensions with niche breadth levels of 2 are greater than or equal to 3.

The second major path is the characteristic development path.

- Agriculturally featured development path:
  The niche suitability level of the sub-dimensional niche of agricultural resources is greater than or equal to 2, the niche overlap level of the sub-dimensional niche of agricultural resources is less than or equal to 3, and the niche breadth level of the sub-dimensional niche of agricultural resources is greater than or equal to 2.
- Industrially featured development path:
  The niche suitability level of the sub-dimensional niche of industrial resources is greater than or equal to 2, the niche overlap level of the sub-dimensional niche of industrial resources is less than or equal to 3, and the niche breadth level of the sub-dimensional niche of industrial resources is greater than or equal to 2.
- Service-featured development path:
  The niche suitability level of the sub-dimensional niche of service resources is greater than or equal to 2, the niche overlap level of the sub-dimensional niche of service resources is less than or equal to 3, and the niche breadth level of the sub-dimensional niche of service resources is greater than or equal to 2.
- Ecological function path:
  The niche suitability level of the sub-dimensional ecological environment resources niche is greater than or equal to 2, the niche overlap level of the sub-dimensional ecological environment resources niche is less than or equal to 3, and the niche breadth level of the sub-dimensional ecological environment resources niche is greater than or equal to 2.

The third major path is the livelihood development path.

- Livelihood development path: The number of niche sub-dimensions with niche suitability levels of 1 are greater than or equal to 6, the number of niche sub-dimensions with niche overlap levels of 1 are less than or equal to 3, and the number of niche sub-dimensions with niche breadth levels of 1 are greater than or equal to 4.

## 3. Results

### 3.1. Niche Suitability Results Analysis

According to the index system of niche suitability evaluation for high-quality development in YRB cities constructed in Table 1 above, the niche suitability evaluation values for the period of 2011–2020 of 7 sub-dimensions of 50 cities in the study basin were calculated according to the method of Section 2.2.1, and the calculation results for 2020 reflecting the current state of niche suitability evaluation in the YRB are shown in Table 2 as follows. The results of the 10-year evaluation will be used later to further measure the niche breadth and niche overlap.

Table 2 shows that most of the YRB cities do not reach the mean value of the niche suitability of each sub-dimension. The niche suitability mean values the 7 sub-dimensions vary widely. Among them, the niche suitability of industrial resources has the highest mean value of 0.3036, while the niche suitability of openness resources has the lowest at 0.0797. This indicates that the niche suitability levels for the high-quality development of the YRB cities vary widely by dimensions.

According to the method in Section 2.2.1, the evaluation values of the niche suitability levels for the high-quality development of YRB cities and the rankings are derived. In this study, the high-quality development of YRB cities is divided into 4 levels. In order to reflect the clustering characteristics of the suitability of YRB cities, “****” means very suitable and is assigned a 4. “***” means relatively suitable and is assigned a 3. “**” means generally suitable and is assigned a 2. “*” means unsuitable and is assigned a value of 1. The niche suitability levels of the 50 cities of 7 dimensions are shown in Table 3.

As can be seen from Table 3, among the 7 niche sub-dimensions for high-quality development, the percentages of cities that are very suitable (“****”) and relatively suitable (“***”) are 10%, 6%, 12%, 10%, 22%, 16%, and 10%, respectively, and the percentages of cities that are not suitable ( “*”) accounted for 52%, 62%, 68%, 70%, 38%, 60%, and 58%, respectively, in the 7 niche sub-dimensions; some cities have outstanding development characteristics. This indicates that YRB cities have different priorities regarding high-quality development. Therefore, the selection of each city’s high-quality development path should first be based on its resource endowment and absolute advantages.

For service industry resources, the proportion of cities that are very suitable (“****”) and relatively suitable (“***”) are the lowest, while the proportion of cities that are not suitable (“*”) is relatively high. This shows that there is a serious division between the two levels in this niche dimension. The development of the service industry is more prominent in cities such as Lanzhou.

For industrial resources, the proportion of cities that are very suitable (“****”) and relatively suitable (“***”) are relatively high, while the proportion of cities that are not suitable (“*”) is the lowest. This niche dimension has the most balanced development among the YRB cities. The industrial developments of cities such as Yulin are more prominent.

For agricultural resources, the proportion of cities that are very suitable (“****”) and relatively suitable (“***”) are relatively high. Among these, agricultural development is more prominent in cities such as Bayannur.

For ecological environmental resources, ecological positions that are very suitable (“****”) and relatively suitable (“***”) are relatively less. Guyuan is more suitable regarding ecological environment resources.

As can be seen from Table 3, among the 50 YRB cities studied, 6 cities (including Wuzhong, Shizuishan, Zhongwei, Pingliang, Wuhai, and Jinzhong) have unsuitable (“*”) niche suitability levels in all 7 sub-dimensions. This indicates that these cities are significantly behind other cities in terms of niche suitability and are in disadvantaged positions in terms of high-quality development throughout the whole basin. Therefore, solving livelihood issues is the primary goal for high-quality development in these cities.

### 3.2. Niche Breadth Results Analysis

The 7 sub-dimensional niche breadth values of the 50 YRB cities from 2011 to 2020 were derived based on Equation (10), and the changes in niche breadth were statistically analyzed based on this, as shown in Table 4.

Table 4 shows that most of the cities in the basin do not reach the mean value of the sub-dimensional niche breadth. In terms of specific cities, the main cities with high resource possessions and utilization levels are the provincial capitals.

In terms of the basins, only more than half of the cities in the industrial resource ecotone and agricultural resource ecotone increased their ecotone width values during the study period, which indicates that the resources available for the high-quality development of the basin as a whole is gradually decreasing.

In the basins, it was only in 2 sub-dimensional niches (i.e., niche of industrial resources and niche of agricultural resources) where the niche breadth values of more than half of the cities increased during the study period. This indicates that the resources available for high-quality development in the YRB (as a whole) are on a decreasing trend. In addition, more than half of the niche breadth values of the cities in service industry resources and infrastructure resources decreased during the study period. This indicates that the competitiveness for the high-quality development in the YRB increased in industrial resources and agricultural resources, while the competitiveness of service resources and infrastructure resources decreased.

**Figure 3:**
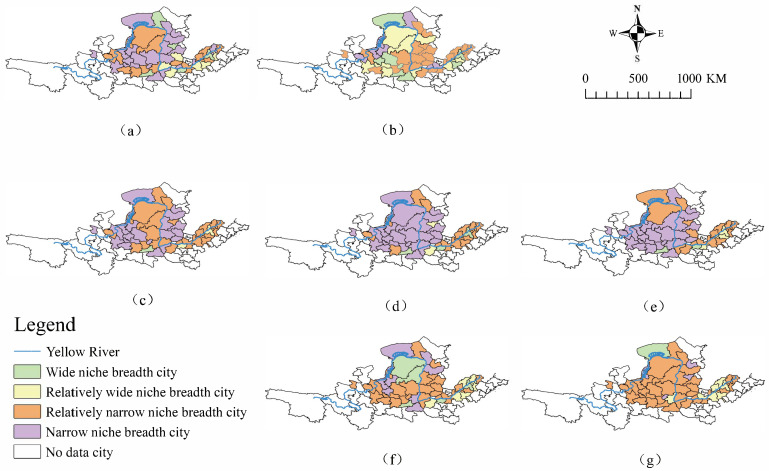
Spatial distribution of the niche breadth in the YRB cities for the period 2011 to 2020: (**a**) Spatial distribution of the niche breadth of infrastructure resources. (**b**) Spatial distribution of the niche breadth of service industry resources. (**c**) Spatial distribution of the niche breadth of openness resources. (**d**) Spatial distribution of the niche breadth of innovative resources. (**e**) Spatial distribution of the niche breadth of industrial resources. (**f**) Spatial distribution of the niche breadth of agricultural resources. (**g**) Spatial distribution of the niche breadth of ecological environment resources.

In order to deeply analyze the spatial distribution patterns of the niche breadth for the high-quality development of YRB cities, this study used ArcGIS software to map the spatial distribution of niche breadth for 50 YRB cities for the period 2011 to 2020, as shown in Figure 3.

From Figure 3, it can be seen that the overall ecological niche breadth values in the YRB are from high to low, i.e., downstream, midstream, and upstream. The spatial distribution of the niche breadth varies significantly among the sub-dimensions.

Specifically, it can be seen from Figure 3a that the niche breadth of infrastructure resources and service industry resources shows an overall trend of “high in the east and low in the west”. Figure 3b shows that the niche breadth of openness resources shows a trend of “low in the south and high in the north” and “low in the west and high in the east”. Figure 3c shows that the niche breadth of innovative resources shows a trend of “high in the east and low in the west”. It can be seen from Figure 3d that the niche breadth of industrial resources shows a trend of “ji” curved central cities and some cities in the downstream. From Figure 3e, the niche breadth of agricultural resources is relatively high in downstream Shandong and Henan and upstream Inner Mongolia. Figure 3f shows that the niche breadth of innovative resources is “high in the east and low in the west”. Moreover, Figure 3g shows that the niche breadth of ecological environment resources shows an overall trend of high in the center and low on both sides of the “ji” bend.

### 3.3. Niche Overlap Results Analysis

**Figure 4:**
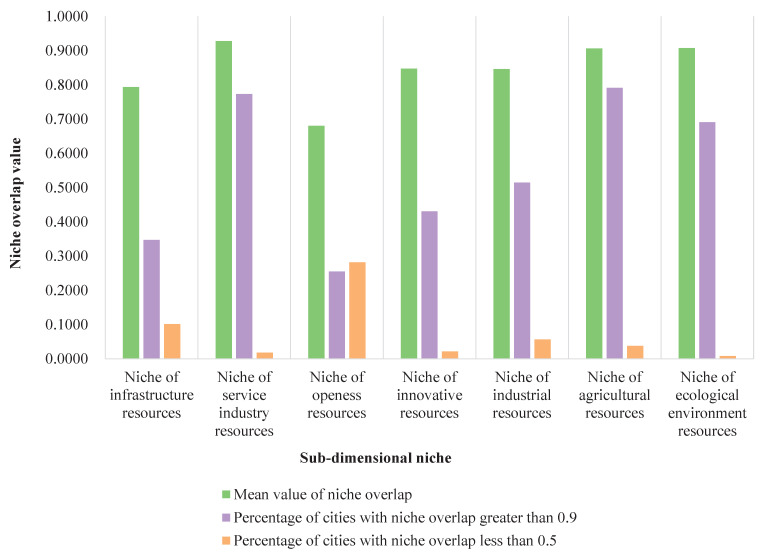
Statistical map of the niche overlaps of YRB cities in 2020.

The sub-dimensional niche overlap values for each YRB city in 2020 were calculated and statistically analyzed according to Equation (11) in Section 2.2.3. The percentages of the number of cities with niche overlaps greater than 0.9 and less than 0.5 among the 7 sub-dimensional niches are shown in Figure 4.

Figure 4 shows that the overlap values of the 7 niche sub-dimensions in the basin are, in descending order: niche of agricultural resource, niche of service resource, niche of ecological and environmental resources, niche of industrial resources, niche of innovative resources, niche of infrastructure resources, and niche of openness resources. Except for openness resources, the mean value of the overlap of the other 6 niches is above 0.7, which indicates that the competition among these 6 niches is relatively intense.

Meanwhile, in terms of spatial distribution, the overlap of ecological niches in the YRB cities (as a whole) goes from high to low in the downstream, midstream, and upstream. Specifically, we analyze the spatial relationship of the overlap degree of ecological niches in 7 sub-dimensions. The niche overlap of infrastructure resources is higher among downstream cities than upstream and midstream cities. The overall niche overlap of service industry resources is the highest in the YRB cities. The niche overlap degree of openness resources is the lowest in the sub-dimensional niches, especially among cities in upstream Gansu Province. Meanwhile, the niche overlap of innovative resources is low in this section of the province and among cities in the midstream. The niche overlap of industrial resources is high, especially between upstream and downstream cities. The niche overlap of agricultural resources and ecological environment resources is high among cities in the downstream.

### 3.4. Suitability Path Selection Analysis

**Figure 5:**
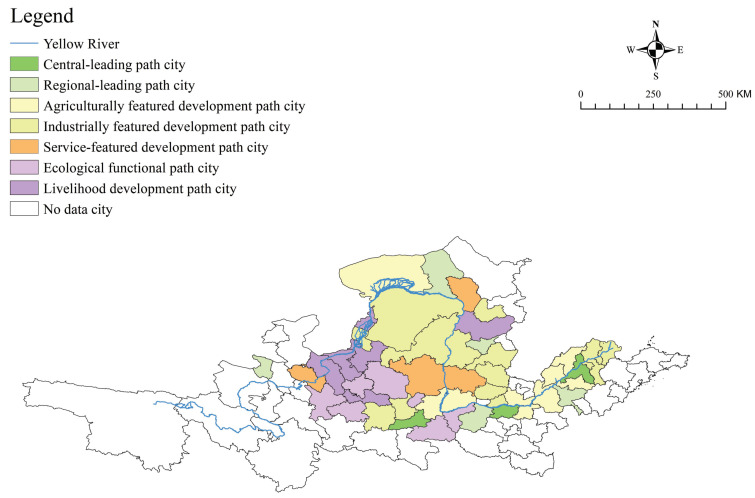
Map of suitability path selection of the high-quality development of YRB cities.

Based on the calculation results of the sub-dimensional niche suitability, niche breadth, and niche overlap of 50 cities, it is clear that the resource endowment for the high-quality development varies widely and unevenly among cities in the YRB. Therefore, to achieve the national strategic goal for high-quality development, the selection of suitability path differs among cities. According to the above analysis results and the path selection method in Section 2.2.4, this paper classifies the suitability paths for the high-quality development of 50 YRB cities. The niche suitability evaluation and niche overlap measurement were selected for the results in 2020 that reflect the current urban development state. Moreover, the niche breadth reflects the result that contains the temporal evolution trend and the potential of the cities, so the average value from 2011 to 2020 is selected. The specific path selection results are shown in Figure 5.

- Leading Development Path

The leading development path is a high-quality development strategy that gives full play to the city’s strengths as a driver. The high-quality development of this city plays a key role in leading and demonstrating the formation of the high-quality development pattern of the YRB. The development of cities suitable for this path is not only outstanding but also relatively balanced in all niche aspects. Therefore, the city selecting this path has a strong development drive and is the most mature core area for urban development in the YRB at this stage. According to the different scopes of influence and radiation, the leading development path is further subdivided into the central-leading path and regional- leading path.

The cities selected for the central-leading path include Xi’an, Zhengzhou, and Jinan. These 3 provincial capitals have much higher levels of development than other cities in their provinces in terms of infrastructure resources, service resources, openness resources, innovative resources, industrial resources, agricultural resources, and ecological environment resources, and they also have greater advantages over other cities throughout the whole basin.

The cities selected for the regional-leading path include Taiyuan, Luoyang, Jining, Baotou, and Xining, which are ranked relatively high among the 50 YRB cities in terms of their high-quality development levels. Although their development levels are slightly lower compared to the central-leading cities, they have absolute advantages in their provinces.

- Characteristic development path

The characteristic development path refers to the development strategy that gives full play to the characteristic industrial advantages of cities and plays a key role in promoting the regional division of labor for the high-quality development of YRB cities. The cities that selected this path are those with significant competitive advantages in a certain sub-dimensional niche in the basin. Compared with the leading developmental cities, the development of these cities is not that balanced, but the characteristic development and regional division of urban labor are also important driving forces for high-quality development. There are 35 cities in this study that were selected for the characteristic development path. According to the differences in advantageous features, they are subdivided into the agriculturally featured development path, industrially feature development path, Service-featured development path, and ecological function path.

There are 10 suitable cities for the agriculturally featured development path, i.e., Bayannur, Weinan, Heze, Dezhou, Liaocheng, Xinxiang, Kaifeng, Tai’an, Yuncheng, and Puyang. As can be seen in Figure 5, these cities are concentrated in the lower reaches of the YRB.

There are 14 suitable cities for the industrially feature development path, i.e., Yulin, Erdos, Binzhou, Zibo, Lvliang, Dongying, Changzhi, Yinchuan, Jiaozuo, Xianyang, Jincheng, Baoji, Shuozhou, and Jinzhong. These cities are concentrated in the middle stream of the YRB.

There are 4 suitable cities for the service-featured development path, i.e., Lanzhou, Yan’an, Hohhot, and Linfen, which are spatially distributed.

There are 7 suitable cities for the ecological function path, i.e., Guyuan, Tianshui, Qingyang, Dingxi, Shangluo, Tongchuan and Sanmenxia. These cities are economically backward and relatively weak in overall development but have high ecological function value.

- The livelihood development path

The livelihood development path is a development strategy that focuses on securing and improving the living and development conditions of low- and middle-income members of society in cities and focuses on improving the living standards of the people.

There are 7 suitable cities for the livelihood development path, i.e., Wuhai, Baiyin, Pingliang, Shizuishan, Zhongwei, Xinzhou, and Wuzhong. These cities are mostly clustered in the upper stream of the YRB. These cities are lagging in the development of each sub-dimensional niche in the basin. On the basis of improving people’s livelihoods, exploring and cultivating special advantageous industries is the focus of the high-quality development of these cities.

## 4. Discussion

Studying the high-quality development of the YRB is a popular topic, but few scholars have used quantitative methods to investigate the high-quality development paths from a theoretical perspective. Therefore, this study introduces the ecological niche theory from ecology to examine the path selection for high-quality development in the YRB. The paper measures the suitability of YRB cities for high-quality development from the perspective of an ecological niche to explore how to choose suitable paths for achieving high-quality development.

Unlike other evaluation systems guided by the “Five Development Concepts” [45,46], this study combines the perspective of ecological niche theory with the actual resources on which the development of watershed cities is based, and constructs a reasonable index system, which makes the research results more relevant to reality and the path selection more scientific and reasonable. Most of the previous research studies only focused on the evaluation of the high-quality development level [47,48]. In contrast, this study shifts the research perspective to the ecological niche. The resource status of each sub-dimension niche is measured in more depth. This makes the study of the high-quality development of the YRB go deeper into the degree of resource possession and the intensity of competition in each sub-dimension. The results show that the polarization for high-quality development in YRB cities is quite obvious. Among them, cities suitable for the leading development path are mostly located in the lower stream of the YRB, while cities suitable for the characteristic development path are mostly located in the middle and lower streams. Moreover, cities suitable for the livelihood development path are basically in the upper stream of the YRB. That is, the high-quality development level in the middle and lower reaches of the YRB is far higher than in the upper reaches. This is consistent with the results of previous studies, in that the high-quality development level in the YRB is lowest in the upper reaches, middle in the middle, and highest in the lower reaches [49,50].

The breadth and overlap of ecological niches in each sub-dimension of cities in the YRB vary greatly, with the most competitive ecological niches for service and industrial resources in mid- and downstream cities. Unlike previous studies that focused on the high-quality development paths of the entire YRB or individual industries or cities, this paper includes all cities in the YRB in the research system.

Although the research results all confirm that there are huge differences in the levels of high-quality development among cities in the upper and middle reaches of the YRB, and that zoning and classification are needed to implement policies, the research perspectives based on the ecological niche theory make the path classification more refined. Most of the previous studies have classified the paths with the help of some main functional areas, and the subsequent development paths are mainly divided into 4 types, i.e., optimized development zones, key development zones, restricted development zones, and prohibited development zones. In contrast, this study classifies the path types based on the resource endowment characteristics derived from the results of the sub-dimensional ecological niche adaptation of the YRB cities, which is more convenient for the basin cities to clarify their directions for high-quality development.

Based on the above analysis and discussion, the following countermeasures are proposed for the implementation strategy of the suitability path for the high-quality development of YRB cities:

1. The cities suitable for the leading development path should first make full use of their own advantages to drive the development of high-quality and all-dimensional niches of neighboring cities, especially to strengthen the leading role in innovation. Second, these cities should focus on improving their “shortcomings”. The suitability of agricultural and ecological environment resources for these cities is relatively low; thus, the rational development and utilization of agricultural and ecological resources should be top priorities. Local authorities should make good use of the city’s location according to local conditions, and at the same time regulate macro policies, to expand the ecological niches of agricultural and ecological environment resources. A typical city, such as Xi’an, while strengthening infrastructure construction, may use advanced manufacturing and electronic information industries and other advantageous industries as carriers, promote the expansion of urban development spaces to the periphery through industrial chain clustering, etc., and smooth the mechanisms of sharing and cooperation with other regions in terms of knowledge and technology, so as to improve the level of industrial development in the peripheral regions. In addition, in terms of ecological environment construction, ecological restoration, improvement of energy utilization, and transformation of green lifestyles can be promoted to enable the sustainable improvement of ecological environment quality;
2. The cities that are suitable for characteristic development paths should first make their characteristic industries bigger, stronger, and more refined to form their own barriers to advantageous development. Through the specialization strategy, the development and utilization of the city’s regional advantages and resource advantages can be specialized and divided. This helps to reach the local monopoly of the city in the sub-dimensional niche of being advantageous in a short period of time, thus forming a natural advantage. Second, these cities should expand the number of niche resources and enhance the suitability of new sub-dimensional niches. At the same time, it ought to mitigate the phenomenon of vicious competition for resources caused by niche overlapping in similar characteristic development cities. For example, Erdos promotes the development of a new energy industry and expands the proportion of high-tech industries, such as energy-saving, environmental protection, and new materials in the industrial system. Meanwhile, it can develop a high-quality cultural tourism industry by relying on its historical and geomorphological features.
3. Cities that choose the path of livelihood development should first improve their livelihoods to raise their living standards. In addition, these cities should actively open up to the outside world and reach a resource-parasitic relationship with other advantageous cities. The focus of this path is to implement ecological location specialization strategy. Cities should accurately locate their own advantageous development points. First, they should improve the suitability of certain resource niches in the region, drive the improvement of the suitability of other resource niches, and finally realize high-quality comprehensive development. The local government will transform disadvantages into advantages by increasing investments, so as to realize the transformation of resource advantages into economic advantages. A typical city, such as Wuhai, should increase the local government’s investment and use the city’s special topography to focus on developing international ecotourism, so as to realize the transformation of resource advantages into economic advantages and promote the improvement of the city’s livelihood in multiple directions.

The current research is a path study based on the resource endowment and high-quality development status of the YRB cities themselves, without considering the cooperation between the basin cities, which have certain limitations. The subsequent study should add relevant elements to enhance the synergy of the high-quality development paths of the basin cities.

## 5. Conclusions

This study addresses the lack of theoretical and quantitative research on high-quality development paths of YRB cities by introducing the ecological niche theory. The proposed classification criteria for high-quality development paths provide more scientific and systematic ways for cities to select their suitable paths. An index system for evaluating niche suitability of high-quality development in the YRB was constructed and used to evaluate niche suitability from 2011 to 2020. The study proposes a method for selecting suitable development paths, leading to the following conclusions.

1. In terms of temporal evolution, the niche suitability of the high-quality development of YRB cities decreased during 2011–2020. Infrastructure resources and service industry resources did not change much, while openness to resources and industrial resources increased. In addition, innovative resources, agricultural resources, and ecological environment resources continued to decrease.
2. In terms of spatial evolution, there are relatively few links between cities in the upper YRB and other cities, and the competition is less intense than that between cities in the lower and middle basins; however, ecological environment resources in the upper stream developed. The unevenness of infrastructure resources and service industry resources for the high-quality development of YRB cities are strong; most are centered in provincial capital cities and radiate outward.
3. The suitability paths for the high-quality development of YRB cities can be divided into 3 major categories: (1) the leading development type, including central-leading type and regional-leading type; (2) the characteristic development type, including agriculturally featured development type, industrially featured development type, service-featured development type, and ecological function type; and (3) the livelihood development type.

The current research provides a systematic consideration of the problem of selecting the suitable paths for the high-quality development of basin cities in China. The study of watershed city development problems using the ecological niche theory and targeted suggestions are also significant for the development of watershed cities in other countries.

## Figures and Tables

**Table 1:** Evaluation index system of niche suitability of the high-quality development of YRB cities.

Niche Dimensions	Evaluation Index	Attributes
Niche of infrastructure resources	City-paved road area at year-end (10${}^{4}$ m${}^{2}$)	+
	Education expenditure (CNY million)	+
	Number of licensed (assistant) doctors (person)	+
	Per capita collections of public libraries (copy)	+
	Road passenger volume (10,000 person)	+
Niche of service industry resources	Tertiary industry employees (person)	+
	Gross value of tertiary industry (CNY billion)	+
Niche of openness resources	Amount of foreign capital actually utilized (USD million)	+
	Foreign exchange income from tourism (USD million)	+
	Total imports and exports (USD million)	+
Niche of innovative resources	Science expenditure (CNY million)	+
	Number of invention patents granted (item)	+
	Number of utility model patents granted (item)	+
	Internal expenditure on R&D expenses (CNY billion)	+
Niche of industrial resources	Total output value of industrial enterprises above the scale (CNY million)	+
	Total fixed assets of industrial enterprises above the scale (CNY million)	+
	Comprehensive utilization rate of industrial solid waste (%)	+
Niche of agricultural resources	Total power of agricultural machinery (million kilowatts)	+
	Number of people employed in agriculture, forestry, animal husbandry and fishery (10,000 person)	+
	Total output value of agriculture, forestry, animal husbandry and fishery (CNY billion)	+
Niche of ecological environment resources	Urban domestic sewage treatment rate (%)	+
	Industrial wastewater discharge (million tons)	−
	Total water resources (10${}^{4}$ m${}^{3}$)	+
	Amount of energy consumption per CNY million GDP (tons of standard coal per CNY million)	−
	Greening coverage of built-up areas (%)	+

**Table 2:** Evaluation values of sub-dimensional niche suitability of the high-quality development of YRB cities in 2020.

Cities	Niche of Infrastructure Resources	Niche of Service Industry Resources	Niche of Openness Resources	Niche of Innovative Resources	Niche of Industrial Resources	Niche of Agricultural Resources	Niche of Ecological Environment Resources
Xining	0.0964	0.0555	0.0165	0.0223	0.2522	0.0678	0.1237
Yinchuan	0.1628	0.0716	0.0170	0.0681	0.3092	0.0999	0.0847
Shizuishan	0.0869	0.0038	0.0048	0.0221	0.1303	0.0363	0.0997
Wuzhong	0.0871	0.0064	0.0022	0.0167	0.1666	0.0787	0.0979
Guyuan	0.0713	0.0023	0.0023	0.0065	0.1696	0.0859	0.5472
Zhongwei	0.0521	0.0003	0.0076	0.0122	0.1038	0.0807	0.1052
Lanzhou	0.1733	0.1551	0.0167	0.1238	0.2649	0.1322	0.0959
Baiyin	0.1513	0.0124	0.0078	0.0104	0.1860	0.1038	0.1174
Tianshui	0.1170	0.0284	0.0049	0.0120	0.2149	0.1030	0.1948
Pingliang	0.0892	0.0175	0.0015	0.0034	0.1915	0.0805	0.1506
Qingyang	0.0842	0.0219	0.0033	0.0050	0.2488	0.0823	0.3014
Dingxi	0.0914	0.0194	0.0014	0.0053	0.2184	0.0830	0.2369
huhehaote	0.1330	0.1323	0.0622	0.0685	0.2593	0.1109	0.1250
Baotou	0.3974	0.1020	0.0303	0.0634	0.3110	0.0763	0.1056
Wuhai	0.0940	0.0010	0.0120	0.0111	0.1579	0.0024	0.0956
Erdos	0.1541	0.0943	0.0659	0.0560	0.7157	0.1120	0.2130
Bayannur	0.0567	0.0244	0.0278	0.0078	0.1030	0.7613	0.3406
Xi’an	0.6448	0.9486	0.7096	0.7219	0.6529	0.2142	0.2198
Tongchuan	0.0689	0.0046	0.0050	0.0022	0.2194	0.0219	0.4133
Baoji	0.1686	0.0603	0.0176	0.0522	0.2420	0.1816	0.1997
Xianyang	0.1748	0.0688	0.0206	0.0363	0.2875	0.2191	0.1396
Weinan	0.1580	0.0690	0.0040	0.0241	0.1895	0.3173	0.1404
Yan’an	0.0940	0.1422	0.0133	0.0210	0.2660	0.1537	0.1691
Yulin	0.1542	0.1049	0.0072	0.0338	0.8510	0.1967	0.1986
Shangluo	0.0756	0.0190	0.0036	0.0049	0.1385	0.0717	0.3382
Taiyuan	0.5766	0.2051	0.1326	0.2732	0.2836	0.0312	0.1419
Changzhi	0.1972	0.0581	0.0286	0.0253	0.3119	0.0795	0.1476
Jincheng	0.1349	0.0377	0.0230	0.0246	0.2752	0.0565	0.1239
Shuozhou	0.0534	0.0424	0.0069	0.0035	0.2293	0.0870	0.1639
Jinzhong	0.1115	0.0511	0.0208	0.0283	0.1944	0.0873	0.1305
Yuncheng	0.2420	0.0659	0.0075	0.0319	0.1916	0.1981	0.1236
Xinzhou	0.0699	0.0364	0.0048	0.0070	0.1758	0.0905	0.1729
Linfen	0.1161	0.0593	0.0022	0.0190	0.2448	0.1037	0.1282
Lvliang	0.0875	0.0444	0.0216	0.0139	0.3447	0.0724	0.1466
Zhengzhou	0.4026	0.4896	0.7922	0.6229	0.6772	0.1615	0.1556
Kaifeng	0.1372	0.0756	0.2755	0.0648	0.2877	0.2457	0.1799
Luoyang	0.2525	0.2430	0.1925	0.2426	0.4219	0.2131	0.1831
xinxiang	0.1769	0.1094	0.0841	0.1200	0.2835	0.2654	0.1374
Jiaozuo	0.0900	0.0742	0.0661	0.0787	0.2855	0.1144	0.1220
Puyang	0.1100	0.0542	0.0494	0.0588	0.2083	0.1703	0.1325
Sanmenxia	0.0829	0.0342	0.0832	0.0393	0.1922	0.0936	0.1769
Jinan	0.4452	0.4156	0.2735	0.5598	0.5682	0.2543	0.2208
Zibo	0.2434	0.1256	0.1033	0.1564	0.4077	0.1146	0.1486
Dongying	0.1516	0.0749	0.1573	0.1070	0.3168	0.1210	0.1165
Jining	0.2833	0.1433	0.0842	0.1437	0.4678	0.3521	0.2272
Taian	0.1646	0.0979	0.2624	0.0893	0.3152	0.2136	0.2031
Dezhou	0.1622	0.1055	0.0478	0.1286	0.3826	0.3484	0.1365
Liaocheng	0.1681	0.0893	0.0503	0.0921	0.3328	0.3029	0.1141
Binzhou	0.1222	0.0856	0.0950	0.1517	0.4831	0.1904	0.1197
Heze	0.2136	0.1314	0.0533	0.0654	0.4463	0.3121	0.1938

**Table 3:** Evaluation results of sub-dimensional niche suitability of the high-quality development in 2020.

Cities	Niche of Infrastructure Resources	Niche of Service Industry Resources	Niche of Openness Resources	Niche of Innovative Resources	Niche of Industrial Resources	Niche of Agricultural Resources	Niche of Ecological Environment Resources
Xining	*	*	*	*	**	*	*
Yinchuan	**	*	*	*	**	*	*
Shizuishan	*	*	*	*	*	*	*
Wuzhong	*	*	*	*	*	*	*
Guyuan	*	*	*	*	*	*	****
Zhongwei	*	*	*	*	*	*	*
Lanzhou	**	***	*	**	**	*	*
Baiyin	**	*	*	*	*	*	*
Tianshui	*	*	*	*	*	*	**
Pingliang	*	*	*	*	*	*	*
Qingyang	*	*	*	*	**	*	***
Dingxi	*	*	*	*	*	*	**
huhehaote	*	**	**	*	**	*	*
Baotou	***	**	*	*	**	*	*
Wuhai	*	*	*	*	*	*	*
Erdos	**	**	**	*	****	*	**
Bayannur	*	*	*	*	*	****	***
Xi’an	****	****	****	****	****	**	**
Tongchuan	*	*	*	*	*	*	***
Baoji	**	*	*	*	**	**	**
Xianyang	**	*	*	*	**	**	*
Weinan	**	*	*	*	*	***	*
Yan’an	*	**	*	*	**	**	**
Yulin	**	**	*	*	****	**	**
Shangluo	*	*	*	*	*	*	***
Taiyuan	****	**	**	***	**	*	*
Changzhi	**	*	*	*	**	*	*
Jincheng	*	*	*	*	**	*	*
Shuozhou	*	*	*	*	*	*	**
Jinzhong	*	*	*	*	*	*	*
Yuncheng	**	*	*	*	*	**	*
Xinzhou	*	*	*	*	*	*	**
Linfen	*	*	*	*	**	*	*
Lvliang	*	*	*	*	**	*	*
Zhengzhou	***	***	****	****	****	**	**
Kaifeng	*	*	***	*	**	**	**
Luoyang	**	**	***	***	***	**	**
xinxiang	**	**	**	**	**	***	*
Jiaozuo	*	*	**	**	**	*	*
Puyang	*	*	*	*	*	**	*
Sanmenxia	*	*	**	*	*	*	**
Jinan	***	***	***	****	***	***	**
Zibo	**	**	**	**	***	*	*
Dongying	**	*	**	**	**	*	*
Jining	**	**	**	**	***	***	**
Taian	**	**	***	**	**	**	**
Dezhou	**	**	*	**	***	***	*
Liaocheng	**	**	*	**	**	***	*
Binzhou	*	**	**	**	***	**	*
Heze	**	**	*	*	***	***	**

**Table 4:** Changes in sub-dimensional niche breadth of the high-quality development of YRB cities from 2011 to 2020.

Sub-Dimensional Niche	Number of Cities with Essentially Constant Niche Breadth Values	Number of Cities with Reduced Niche Breadth Values	Number of Cities with Increased Niche Breadth Values
Niche of infrastructure resources	9	28	13
Niche of service industry resources	5	36	9
Niche of openness resources	7	19	24
Niche of innovative resources	8	20	22
Niche of industrial resources	5	19	26
Niche of agricultural resources	5	19	26
Niche of ecological environment resources	6	25	19

## Data Availability

Not applicable.

## Abbreviations

The following abbreviations are used in this manuscript:

YRB	Yellow River basin
